# Role of transient receptor potential (TRP) channels in osteoarthritis: a comprehensive review

**DOI:** 10.3389/fphar.2025.1711074

**Published:** 2026-01-15

**Authors:** Pengyan Qiao, Wei Wang, Sumiao Liu, Yanli Yang, Pingzhi Wang, Yang Liu, Yazhen Su, Peng Hu, Jie Pan, Liyun Zhang

**Affiliations:** 1 Shanxi Bethune Hospital, Shanxi Academy of Medical Sciences, Tongji Shanxi Hospital, Third Hospital of Shanxi Medical University, Taiyuan, Shanxi, China; 2 Department of Laboratory Medicine, Shanxi Provincial People’s Hospital, Taiyuan, China; 3 Department of Rheumatology, Shanxi Bethune Hospital, Shanxi Medical University, Taiyuan, China; 4 Department of Pathology, Stanford University School of Medicine, Palo Alto, CA, United States

**Keywords:** chondrocytes, osteoarthritis, pain, synovial inflammation, TRP channels

## Abstract

Osteoarthritis (OA) is a chronic degenerative joint disease, primarily characterized by the degeneration of articular cartilage, synovial inflammation, and persistent pain, which severely impairs the quality of life for hundreds of millions of patients worldwide. Transient receptor potential (TRP) channels, a group of non-selective cation channels activated by various physicochemical stimuli, play a crucial role in the pathogenesis of OA. This review systematically explores the roles of the different TRP channel subfamilies, including TRPV, TRPA, TRPC, and TRPM, in OA-affected joint tissues. It highlights how TRP channels contribute to cartilage degradation and synovitis through multiple mechanisms, including the modulation of intracellular calcium signaling, the regulation of inflammatory responses, and the control of chondrocyte metabolism, apoptosis, and ferroptosis. Additionally, the critical role of TRP channels as molecular sensors of pain is discussed in detail. These channels have been shown to both mediate the initiation and transmission of nociceptive signals in sensory neurons, and to enhance pain sensitivity through interactions with immune cells. Consequently, targeting TRP channels with specific agonists or antagonists has emerged as a promising strategy for developing novel analgesics. This review outlines recent clinical progress and the therapeutic promise of targeting the TRP channel network for OA pain relief and disease modification.

## 1 Introduction

**FIGURE 1:**
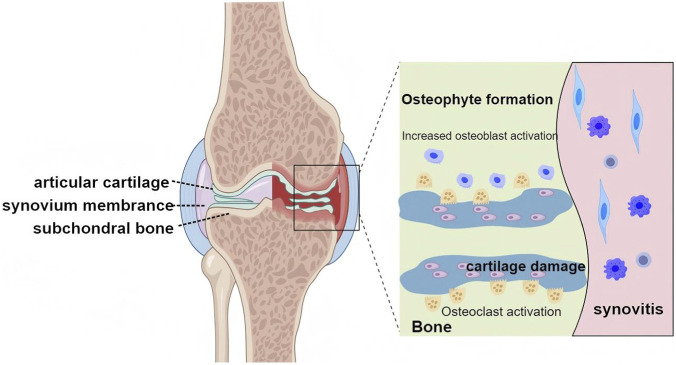
Pathology of OA.

Osteoarthritis (OA) is the most prevalent form of arthritis, affecting approximately 655 million people worldwide, and it is the leading cause of disability in the elderly (Schafer and Grassel, 2022). Already a significant burden on healthcare systems globally, the prevalence of OA is only increasing (Schafer and Grassel, 2022). This disease is characterized by the degeneration of articular cartilage, subchondral bone sclerosis, synovial inflammation, and osteophyte formation, which collectively contribute to joint dysfunction and pain (Mahmoudian et al., 2021; Yu et al., 2022; Peat and Thomas, 2021; Huang et al., 2018). The multifactorial etiology of OA involves a combination of genetic, biomechanical, and environmental factors, including age, joint injury, obesity, and sex, as it predominantly affects females (Quicke et al., 2022; Kim et al., 2023; Grassel et al., 2021). Despite substantial research into OA pathophysiology, the complex mechanisms underpinning disease progression, particularly the roles of ion channels and signaling pathways, remain incompletely understood (Figure 1).

The central symptom of OA is pain, and this arises from a combination of inflammatory, mechanical, and nociceptive factors (Yu et al., 2022; Yao et al., 2023). Peripheral sensory neurons are abundant in synovial tissues, cartilage, and subchondral bone, and they detect harmful stimuli such as tissue damage and inflammation, initiating pain signals that are relayed through the dorsal root ganglia (DRG) to the spinal cord. These signals are further processed in the dorsal horn of the spinal cord and transmitted to the brain, where pain perception occurs (Fu et al., 2024; Vincent, 2020; Gonçalves et al., 2022).

Ion channels, particularly transient receptor potential (TRP) channels, have emerged as crucial mediators in OA pathogenesis (Zhou et al., 2024). Members of this mechanosensitive channel family are activated by various different stimuli, including pressure, shear stress, and tension, and several have been reported to play an essential role in maintaining joint function (Pei et al., 2021). Because of their pivotal roles in mechanosensation and their reported roles in OA pathogenesis, several channels in the TRP family have been extensively studied (Benítez-Angeles et al., 2020; Kim, 2018; Sasase et al., 2022; Nummenmaa et al., 2016; Hinata et al., 2018; Xing et al., 2017).

## 2 TRP ion channels are versatile sensors in cellular physiology

The discovery of TRP channels dates back to 1969, when Cosens and collaborators identified visual mutants in fruit flies that exhibited abnormal light responses, spawning the term “transient receptor potential” (TRP) (Cosens and Manning, 1969). Over subsequent years, significant advancements in sequencing technologies have facilitated the characterization of TRP channels across various species, including many mammal species, where they have been shown to play crucial roles in numerous physiological processes (Minke et al., 1975; Zhang et al., 2023).

In mammals, the TRP channel superfamily comprises six subtypes: transient receptor potential canonical (TRPC), transient receptor potential ankyrin (TRPA), transient receptor potential vanilloid (TRPV), transient receptor potential melastatin (TRPM), transient receptor potential mucolipin (TRPML), and transient receptor potential polycystin (TRPP). These subtypes encompass 28 subfamilies, including TRPC1-7, TRPV1-6, TRPM1-8, TRPML1-3, TRPP2/3/5, and TRPA1. Remarkably, these nonselective permeable cation channels serve as versatile sensors, detecting temperature variation, inflammatory signals, pain cues, and chemical substances, among other functions. In 2021, David Julius and Ardem Patapoutian received the Nobel Prize in Physiology or Medicine for their groundbreaking discoveries on temperature and touch receptors, including on TRP channels and PIEZO receptors (Prize announcement, 2021).

Structurally, TRP channels consist of six membrane-spanning domains (S1–S6), with a pore-forming loop between S5 and S6 (Zhang et al., 2023). Because of this arrangement, the C-terminus and N-terminus are both situated intracellularly. In general, TRP channels act as ‘sentinel’ molecules, sensing diverse physical and chemical stimuli, including temperature fluctuations, pH changes, inflammation, metabolites, osmotic pressure, and mechanical stress, and then orchestrating the appropriate cellular responses. Many of these channels have been reported to play pivotal roles in signal transduction, cellular survival, and adaptation to environmental cues, underscoring their importance in cellular homeostasis (Zhang et al., 2023; Wu et al., 2010; Peier et al., 2002).

In patients with OA, several TRP channels have been reported to play crucial roles in perceiving and transmitting noxious physiological stimuli such as pain, temperature, and inflammation. Many other studies have demonstrated the presence of various subtypes of TRP channels on cartilage and synovial cells, revealing their potential association with the mechanisms underlying inflammation and pain.

## 3 Role of TRP channels in OA pathogenesis: non-neuronal cells

### 3.1 TRP channels in OA chondrocytes: roles and mechanisms

**FIGURE 2:**
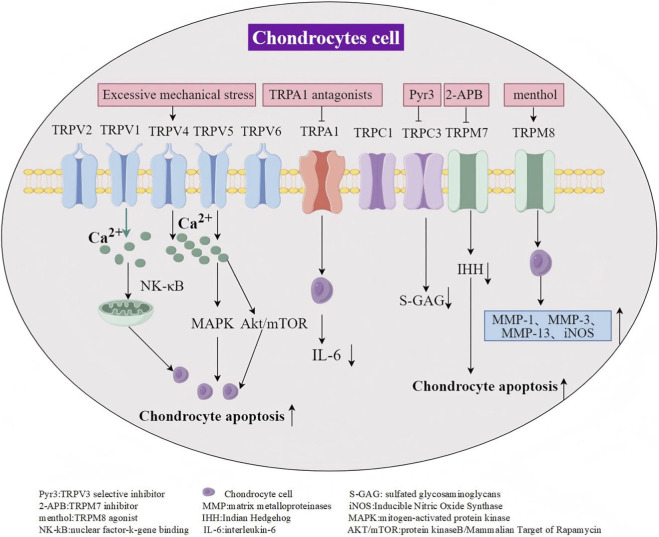
TRP channels in OA chondrocytes.

Chondrocytes, specialized cells found exclusively in articular cartilage, play a crucial role in maintaining the normal function of cartilage. Moreover, dysfunctional cellular processes within cartilage contribute to OA development (Coryell et al., 2021). Because of their impact on cartilage homeostasis, the roles of the TRP channels expressed in chondrocytes in these dysfunctional processes has drawn significant interest (Figure 2; Table 1).

**TABLE 1:** Roles of TRP subtypes in osteoarthritis articular cartilage.

TRP subtype	Model	Expression	Potential mechanism	References
TRPV1	OA patient chondrocytes, IL-1 induced chondrocytes	Increased	TRPV1 gene expression is also elevated in freshly isolated OA chondrocytes, and in chondrocytes stimulated with IL-1 and TNF-α TRPV1 agonists activate TRPV1 channels in OA chondrocytes, promoting GPX4 expression, reducing ferroptosis, and inhibiting chondrocyte degradation	Gavenis et al. (2008), Valdes et al. (2011), Charlier et al. (2016), Lv et al. (2022), Miao et al. (2022), Ohtsuki et al. (2019)
TRPV2	OA patients, mouse joint cartilage	Increased	TRPV2 is expressed in both mouse and human articular cartilage, and also in ectopic ossification lesions	Nakamoto et al. (2021)
TRPV4	ALCT joint cartilage	Increased	Excessive mechanical stress induces chondrocyte apoptosis via TRPV4-mediated intracellular Ca^2+^ influx	Xu et al. (2019)
TRPV5	MIA	Increased	TRPV5 activates CaMKII phosphorylation via Ca^2+^ influx, activating MAPK and Akt/mTOR pathways to affect chondrocyte apoptosis	Wei et al. (2018), Eid and Ito (2021)
TRPV6	Human joint chondrocyte culture (P0), horse joint cartilage	Expressed	TRPV6 deficiency affects chondrocyte functions such as extracellular matrix secretion, matrix metalloproteinase release, cell proliferation, and apoptosis	Song et al. (2017)
TRPA1	MIA chondrocytes	Increased	Treatment with TRPA1 antagonists significantly downregulates IL-6 expression in chondrocytes	Moilanen et al. (2015), Li et al. (2021), Nummenmaa et al. (2021)
TRPC1	OA chondrocytes	Increased	NA	Halonen et al. (2023)
TRPC3	Human joint chondrocyte culture (P2)	Increased	Inhibition of TRPC3 reduces S-GAG content	Gavenis et al. (2008), Savadipour et al. (2021)
TRPM7	Human joint chondrocyte culture, MIA	Increased	Blocking TRPM7 reduces apoptosis in MIA-induced rat joint chondrocytes and cartilage injury	Gavenis et al. (2008), Ma et al. (2021)
TRPM8	OA patient chondrocytes, AIA	Increased	Decreased TRPM8 expression on chondrocytes after dexamethasone intervention following pro-inflammatory cytokine IL-1β stimulation	Halonen et al. (2023), Bin et al. (2014)

#### 3.1.1 Overview of the roles and expression patterns of TRPV family channels in OA chondrocytes

##### 3.1.1.1 TRPV1

The expression of TRPV1, which is implicated in cartilage homeostasis, is increased in OA chondrocytes. TRPV1 gene expression is also elevated in freshly isolated OA chondrocytes, and in chondrocytes stimulated with IL-1 and TNF-α. The observed changes in chondrocyte TRPV1 expression levels contribute significantly to the structural damage associated with OA (Gavenis et al., 2008; Valdes et al., 2011).

Various forms of chondrocyte death, including necrosis, apoptosis, autophagic cell death, and ferroptosis, are involved in the pathogenesis of OA (Valdes et al., 2011; Charlier et al., 2016; Lv et al., 2022). Research suggests that TRPV1 agonists, such as capsaicin, can activate TRPV1 channels in OA chondrocytes, promoting the expression of the antioxidant enzyme glutathione peroxidase 4 (GPX4). GPX4 is a crucial regulator of ferroptosis, and it may protect chondrocytes from the effects of iron overload, reduce cartilage degradation, and decrease susceptibility to oxidative stress and extracellular matrix degradation (Miao et al., 2022). These findings imply a multifaceted role for TRPV1 in sustaining cartilage homeostasis, warranting further investigation into its mechanisms in OA and its impact on chondrocyte function.

Additionally, TRPV1 mediates the chondroprotective effects of cyclic tensile strain by suppressing cytokine-induced calcium influx and NF-κB activation.While cytokine stimulation induced an increase in intracellular Ca^2+^ concentration, downstream translocation of nuclear factor-kappa B (NF-κB), and extracellular matrix degradation, mechanical strain-induced activation of TRPV1 channels caused Ca^2+^ efflux, which blocked the cytokine-stimulated translocation of nuclear NF-κB from the cytoplasm to the nucleus (Ohtsuki et al., 2019).

##### 3.1.1.2 TRPV2

TRPV2 is expressed in both mouse and human articular cartilage, and also in ectopic ossification lesions (Nakamoto et al., 2021). A *Trpv2*-KO mouse model (specifically, chondrocyte-specific *Trpv2*-flox mice) exhibited increased articular cartilage degradation, reduced expression of *lubricin*/*Prg4* (which encodes Proteoglycan 4, a protein essential for maintaining lubrication of the joint surface), and significant ectopic ossification around the joints. Additional analyses revealed that mechanical stress-dependent TRPV2 signaling (and subsequent Ca^2+^ influx) induced *Prg4* expression via the Ca^2+^/calmodulin-dependent protein kinase-cAMP response element-binding protein axis. In *Trpv2*-knockout mouse chondrocytes, induction of *Prg4* expression by fluid flow-induced shear stress was abolished. Hence, TRPV2 plays a role in regulating articular cartilage through *Prg4* induction and suppression of ectopic ossification.

##### 3.1.1.3 TRPV4

TRPV4 was upregulated in the articular cartilage of an anterior cruciate ligament transection (ACLT)-induced OA rat model (Xu et al., 2019), and excessive mechanical stress triggered chondrocyte apoptosis via TRPV4-mediated Ca^2+^ influx. However, treatment with TRPV4 inhibitors was observed to mitigate the resulting cartilage degeneration. These results highlight the potential of TRPV4 as a drug target for OA treatment.

In a rat model of knee OA, intragastric administration of Iguratimod (IGU), a small molecule anti-rheumatic drug with a protective effect on cartilage, increased TRPV4 expression, leading to significant pain relief and the inhibition of cartilage destruction (Wang L. et al., 2023). Moreover, *in vitro* experiments using cultured chondrocytes revealed that IGU intervention enhanced rat cartilage differentiation, activity, and migration. Hence, IGU plays a role in slowing cartilage destruction and promoting cartilage differentiation and migration, potentially through the TRPV4 ion channel pathway.

##### 3.1.1.4 TRPV5

In a monosodium iodoacetate (MIA)-induced OA model, TRPV5 expression was reportedly upregulated in cartilage (Wei et al., 2018). The consequent elevation in TRPV5 activity was observed to activate calcium/calmodulin-dependent protein kinase II (CaMKII) phosphorylation (via Ca^2+^ influx). Activated p-CaMKII is known to play a crucial role in chondrocyte apoptosis—the most common pathological feature in OA cartilage—via the MAPK and Akt/mTOR pathways. Interestingly, oxoglaucine was reported to alleviate OA by blocking TRPV5-mediated Ca^2+^ influx, thereby reducing calmodulin/CAMK-II pathway activity, and ultimately activating autophagy (Eid and Ito, 2021).

##### 3.1.1.5 TRPV6

TRPV6 is expressed in acute primary cultures of human articular chondrocytes at passage 0 (P0), although TRPV6 expression is absent by passage 2 (P2) cells (Coryell et al., 2021). In the knee articular cartilage of OA rats and OA patients, TRPV6 expression was notably reduced (in comparison with control levels). Hdudet et al. also confirmed the presence of TRPV6 channels in horse articular cartilage cells (Hdud et al., 2012). Gene knockout of TRPV6 in mice exacerbated osteoarthritic changes, including cartilage destruction, effusion, and loss of proteoglycans. Moreover, the absence of TRPV6 significantly impacted chondrocyte functions, including extracellular matrix secretion, the release of matrix-degrading enzymes, chondrocyte proliferation, and chondrocyte apoptosis (Song et al., 2017). Hence, TRPV6 channels function as chondroprotective factors with a significant role in the pathogenesis of OA.

#### 3.1.2 Role of TRPA1 in OA chondrocytes

Chondrocyte TRPA1 expression was increased in an MIA mouse model of OA (Moilanen et al., 2015). Interestingly, the expected knee joint changes were less severe in TRPA1-deficient mice after MIA injection. Additional research utilizing cartilage or cartilage cells from wild-type (WT) and TRPA1 knockout (KO) mice, and primary chondrocytes from OA patients, has revealed that TRPA1 deficiency significantly decreased the expression of IL-6 family cytokines, including leukemia inhibitory factor (LIF) and IL-11. Moreover, treatment with TRPA1 antagonists markedly reduced the expression of IL-6 in chondrocytes, thereby inhibiting inflammation (Li et al., 2021; Nummenmaa et al., 2021).

#### 3.1.3 Role of TRPC subtypes in OA chondrocytes

##### 3.1.3.1 TRPC1

A next-generation sequencing (NGS) analysis of TRP gene expression revealed that the expression levels of 19 TRP genes, including *TRPM7*, *TRPV4*, *TRPC1*, and *TRPM8*, were significantly elevated in chondrocytes (Halonen et al., 2023). Furthermore, *TRPC1* expression was not significantly changed after IL-1β-induced chondrocytes were treated with dexamethasone or ibuprofen. In cultured human knee femoral condyle chondrocytes, *TRPC1* expression, as measured using real-time fluorescence quantitative PCR (RT‒qPCR), was similar in P0 and P2 (passage 0, P0; passage 2, P2) cells (Gavenis et al., 2008).

##### 3.1.3.2 TRPC3

Studies investigating the mechanobiology of chondrocytes exposed to static hydrostatic pressure revealed that inhibition of TRPC3 and TRPV4 reduced the content of sulfated glycosaminoglycan (S-GAG) (Savadipour et al., 2021). Additionally, RT‒qPCR analysis of human knee femoral condyle chondrocytes revealed elevated *TRPC3* and *TRPC6* gene expression levels in passage 2 (P2) cells (Gavenis et al., 2008).

#### 3.1.4 Role of TRPM subtypes in OA chondrocytes

##### 3.1.4.1 TRPM7

RT‒qPCR analysis revealed that *TRPM7* gene expression was increased in cultured human knee femoral condyle chondrocytes (Gavenis et al., 2008). In a modified Hulth method-induced osteoarthritic mouse model, apigenin alleviated cartilage injury by reducing TRPM7 and p-mTOR protein levels in macrophages, while significantly increasing the level of Bcl2 protein (Ji et al., 2023). Moreover, apigenin also decreased the phosphorylation levels of IL-1, IL-6, MMP13, TNF-α, P38, JNK, and ERK in chondrocytes. These findings suggest that apigenin mitigates inflammation induced by macrophage polarization and chondrocyte apoptosis in a macrophage-chondrocyte co-culture system through the TRPM7‒mTOR pathway. In rats, blocking TRPM7 reduced the joint chondrocyte apoptosis and cartilage injury caused by MIA (Ma et al., 2021). TRPM7 inhibitors can also suppress TRPM7 channels and the IHH signalling pathway *in vivo* to protect cartilage.

##### 3.1.4.2 TRPM8

Yubin et al. reported that TRPM8 expression on the surface of cartilage was significantly higher in OA patients (compared with normal individuals) (Bin et al., 2014). While NGS analysis provided confirmation that *TRPM8* expression was elevated in OA chondrocytes (Gavenis et al., 2008; Nummenmaa et al., 2021), subsequent IL-1β stimulation of OA chondrocytes decreased *TRPM8* expression. Additionally, RT‒qPCR analysis revealed that *TRPM7*, *TRPV4*, *TRPC1*, and *TRPM8* gene expression levels were increased in chondrocytes. Interestingly, treatment with dexamethasone was reported to reduce *TRPM8* expression in IL-1β-stimulated chondrocytes (Halonen et al., 2023).

### 3.2 TRP channels in OA synovial cells: roles and mechanisms

**FIGURE 3:**
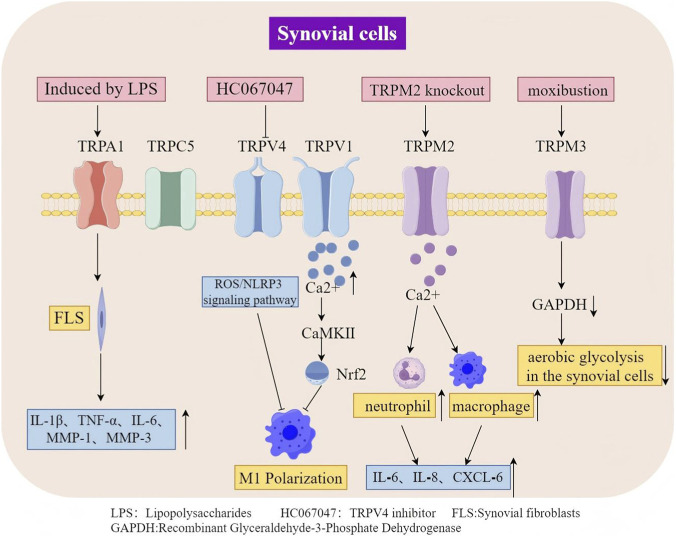
TRP channels in OA Synovial Cells: Roles and Mechanisms.

In synovial joints, synovial cells (or “synoviocytes”) line the capsule and form the synovial membrane. These synovial cells are important for nourishing the tissues, joint lubrication, and immune functions in OA. They can be classified as macrophage-like synoviocytes, fibroblast-like synoviocytes, or mesenchymal stem cells (MSCs). Physiologically, they maintain joint homeostasis and healthy joint function by providing lubrication, immune surveillance, and a rapid response to noxious injury. Synovial fibroblasts and macrophages secrete pro-inflammatory cytokines (e.g., IL-1β,TNF-α, IL-6) and matrix-degrading enzymes (MMPs, ADAMTS), driving synovitis, cartilage destruction, and pain. Synovial macrophages can polarize toward a pro-inflammatory M1 phenotype, exacerbating inflammation, while SMSCs possess immunomodulatory and chondrogenic potential that may offer therapeutic benefits. Pathological changes also include synovial hyperplasia, fibrosis, and angiogenesis, which further impair joint function. The crosstalk between synovial cells and chondrocytes via cytokines and extracellular vesicles creates a vicious cycle of inflammation and degradation. Targeting synovial cells presents a promising therapeutic strategy for OA (Zou et al., 2023) (Figure 3; Table 2).

**TABLE 2:** Roles of TRP subtypes in osteoarthritic synovial cells.

TRP subtype	Model	Synovial cell type	Expression	Potential mechanism	References
TRPV1	Human synovial cells, MIA	Synovial M1 macrophages, FLS	Increased	TRPV1 alleviates osteoarthritis by inhibiting M1 macrophage polarization via the Ca^2+^/CaMKII/Nrf2 signalling pathway	Kelly et al. (2015), Lv et al. (2021), Liao et al. (2023)
TRPV4	ALCT articular cartilage	Synovial M1 macrophages	Increased	Inhibition of TRPV4 delays OA progression by inhibiting M1 synovial macrophage polarization via the ROS/NLRP3 pathway	Nguyen et al. (2022)
TRPA1	ALCT, human OA cell cultures	FLS	Increased	The functional expression of TRPA1 was also increased in primary human OA-FLSs induced by LPS.	Yin et al. (2018)
TRPC5	MIA mice	Synovial hypertrophic cells	Increased	Loss of TRPC5 receptor signalling pathway associated with exacerbation of OA pain-like behaviour, which correlates with increased expression of enzymes involved in extracellular remodelling, synovial inflammatory cells, and activation and damage of neurons in the DRG.	Alawi et al. (2017)
TRPM3	OA patients	Synovial cells	Effects on glucose metabolism	Downregulation of GAPDH modulates glucose metabolism in knee joint synovial cells, mediating anti-inflammatory effects	Duitama et al. (2020)

#### 3.2.1 Role of TRPV subtypes in OA synovial cells

##### 3.2.1.1 TRPV1

TRPV1 is significantly expressed in the synovial cells of humans and rats with OA (Kelly et al., 2015; Lv et al., 2021). Moreover, TRPV1 is predominantly expressed in synovial macrophages that express CD68, a marker for these immune cells. Notably, approximately 90% of the M1 macrophages that infiltrate the synovium express TRPV1 (Lv et al., 2021). In a rat OA pain model (the MIA model), intra-articular injection of TRPV1 antagonists effectively inhibited pain, joint swelling, synovitis, cartilage damage, and osteophyte formation (Kelly et al., 2015).

TRPV1 mRNA and protein expression levels were increased in synovial tissues obtained from human OA patients and rat arthritis models (Kelly et al., 2015; Lv et al., 2021). In fibroblast-like synoviocytes (FLSs), TRPV1 activation promoted the expression of proinflammatory cytokines and contributed to the partial generation of reactive oxygen species (ROS) (Liao et al., 2023). In turn, TRPV1 expression can itself be upregulated by proinflammatory cytokines and ROS.

##### 3.2.1.2 TRPV4

TRPV4 expression levels and M1 synovial macrophage infiltration are both increased in OA synovium (Nguyen et al., 2022). In a rat model of OA, intra-articular injection of HC067074, a specific inhibitor of TRPV4, was reported to alleviate OA progression (Sun et al., 2022). TRPV4 inhibition suppressed M1 synovial macrophage polarization via the ROS/NLRP3 pathway, thereby delaying OA progression.

#### 3.2.2 Role of TRPA1 in OA synovial cells

TRPA1 is highly expressed in FLSs in knee OA (KOA) animal models (Song et al., 2017). Local application of Sanse Powder Essential Oil Nanoemulsion (SPNE) gel effectively reduced synovitis in KOA rats by downregulating the mRNA and protein levels of IL-1β, IL-18, and TRPA1 (Li et al., 2021).

The functional expression of TRPA1 was also increased in primary human OA-FLSs induced by LPS (lipopolysaccharide) (Yin et al., 2018). Activation of TRPA1 enhances the inflammatory response, promotes the expression of multiple inflammatory factors, and participates in the regulation of the inflammatory cascade through the Ca^2+^signaling pathway Interestingly, pharmacological inhibition of TRPA1 elicited a protective effect on LPS-induced arthritis.

#### 3.2.3 Role of TRPC subtypes in OA synovial cells

##### 3.2.3.1 TRPC5

TRPC5 is functionally expressed on a range of cells in the synovium of wild-type (WT) mice, including on FLSs. Its activation plays a protective role against inflammatory arthritis, as genetic deletion or pharmacological blockade of TRPC5 exacerbates joint inflammation, pain, and synovitis in murine models (Alawi et al., 2017). In the MIA-induced OA model, genetic deletion of TRPC5 exacerbated pain-like behavior, as evidenced by enhanced mechanical sensitivity. This aggravation of pain was accompanied by marked synovial inflammation, including elevated expression of the mast cell marker CD117, upregulation of extracellular remodeling enzymes, and increased neuronal injury in the DRG. Consistently, across two distinct OA models, loss of TRPC5 signaling was associated with a heightened pain phenotype and activation of pain-related neuroaxis pathways (De Sousa Valente et al., 2020).

#### 3.2.4 Role of TRPM subtypes in OA synovial cells

##### 3.2.4.1 TRPM3

TRPM3 channels are crucial to cellular calcium homeostasis and are implicated in pain perception. Interestingly, moxibustion may contribute to the upregulation of TRPM3 channels in OA (Zhang et al., 2020). Moreover, upregulation of TRPM3 (by moxibustion therapy) was reported to impact calcium metabolism and OA-associated swelling. Additionally, GAPDH was downregulated in synovial cells of the knee joint after moxibustion treatment. Because GAPDH plays a central role in glucose metabolism, its modulation may mediate anti-inflammatory effects.

## 4 TRP channels in OA pain sensing and modulation

TRP channels are a family of non-selective cation channels that are widely expressed across many different cell types. These channels can be activated by a range of physical and chemical stimuli, including temperature, mechanical force, pH changes, osmotic pressure, and various chemical substances (Duitama et al., 2020; Silverman et al., 2020). Multiple subtypes of TRP channels are reportedly expressed in joint tissues, including in chondrocytes, synovial cells, macrophages, and the sensory neurons that innervate the joint (Liao et al., 2023; Matta et al., 2023). These TRP channels help regulate several important pathophysiological processes, including cartilage homeostasis, inflammatory responses, and apoptosis. Furthermore, they play a pivotal role in the generation, transmission, and sensitization of pain signals associated with OA (Liao et al., 2023; Chen et al., 2024).

In sensory neurons, several TRP channel subtypes—specifically TRPV1, TRPA1, and TRPM3—play central roles in the initiation and transmission of pain signals (Matta et al., 2023). In addition to directly exciting nociceptive neurons, TRP channels can indirectly modulate inflammatory responses and pain sensitization through interactions with non-neuronal cells, including immune cells (Duitama et al., 2020). Consequently, TRP channels have emerged as promising targets in the development of novel non-opioid analgesics. For example, high-concentration capsaicin patches are already used clinically in the treatment of neuropathic pain (Silverman et al., 2020). However, given the large number of TRP channel family members and their functional complexity, elucidating their specific mechanisms in different pain states—and assessing their potential and limitations as drug targets—remains a major challenge in pain research. Hence, a comprehensive understanding of the roles of TRP channels in OA pain perception and modulation is essential for the development of novel and effective analgesic therapies (Mobasheri et al., 2024).

### 4.1 The dual role of TRPV1 in OA pain

TRPV1, one of the most extensively studied TRP channels, is primarily a receptor for heat and capsaicin, and it plays a crucial role in pain transmission (Liao et al., 2023; Qu et al., 2023). Multiple studies have confirmed that TRPV1 expression is significantly upregulated in the sensory neurons of OA patients and OA animal models (Kelly et al., 2015; Lv et al., 2021). In a rat OA model, intra-articular injection of the TRPV1-specific agonist capsaicin significantly reduced joint swelling, synovitis, and cartilage damage. The underlying mechanism likely involves TRPV1-dependent Ca^2+^ influx, which subsequently inhibits M1 macrophage polarization through the CaMKII/Nrf2 signaling pathway, thereby exerting anti-inflammatory effects (Lv et al., 2021). However, TRPV1 is also directly involved in pain signal transmission. As a nociceptor on sensory nerve endings, TRPV1 activation leads to the generation of pain signals and central sensitization (Liao et al., 2023). Hence, TRPV1 may play contradictory roles in osteoarthritis, mediating both protective anti-inflammation and direct nociception, effects that are driven by its activation in immune cells and sensory neurons, respectively. This dual functionality positions TRPV1 as a key therapeutic target. However, the development of TRPV1 antagonists is hampered by their significant side effects, which include hyperthermia (Mobasheri et al., 2024; Bamps et al., 2021). This has prompted clinical investigations of alternative agonist-based approaches for pain relief, such as high-dose capsaicin patches and intra-articular resiniferatoxin injections (Mobasheri et al., 2024; Koivisto et al., 2024).

### 4.2 The central role of TRPV4 in OA mechanical pain

TRPV4 is a mechanosensitive ion channel expressed in both chondrocytes and sensory neurons, and it plays a central role in mechanical hyperalgesia in OA (Soga et al., 2021; Kudsi et al., 2024). Deletion of TRPV4 did not affect the pathological progression of OA in an MIA OA model established in *TRPV4* gene knockout (*TRPV4*-KO) rats (Soga et al., 2021). However, loss of TRPV4 activity completely alleviated OA-related mechanical pain behaviors, including reduced grip strength and mechanical hyperalgesia. Furthermore, while electrophysiological recordings revealed an increase in excitability in DRG neurons obtained from wild-type MIA rats (via sensitization through a TRPV4-dependent mechanism), this phenomenon was absent in *TRPV4*-KO rats. These analgesic effects could be recapitulated using a TRPV4 antagonist. Together, these findings suggest that TRPV4 sensitization plays a key role in the development of OA-related mechanical pain. Additionally, TRPV4 antagonists were reported to elicit analgesic effects in various preclinical models of musculoskeletal pain (Kudsi et al., 2024).

### 4.3 The role of TRPA1 in pain

TRPA1 is generally considered a receptor for chemical and cold stimuli, and it is known to play an important role in inflammatory pain. A high-throughput sequencing analysis of human OA chondrocytes revealed that IL-1β significantly upregulated *TRPA1* expression (Halonen et al., 2023). Hence, TRPA1 may play a role in OA inflammation and the cartilage degradation processes. The above evidence points to TRPA1 as a mediator of OA inflammation and pain, particularly pain related to environmental factors, positioning it as an important target for intervention.

### 4.4 TRPC3 and OA pain

TRPC3 has been associated with various chronic pain conditions, including OA, cervical spondylosis, and intervertebral disc herniation. Interestingly, a single-nucleotide polymorphism in the TRPC3 gene, Rs11726196, has a demonstrated association with chronic pain (Xie et al., 2023; Aoki et al., 2023). From these observations, TRPC3 has emerged as a novel target for treating chronic pain (Xia et al., 2015). A downstream signalling cascade involving cGMP-dependent protein kinase I (PKG-I), which is activated by the NMDA receptor-NO-cGMP pathway, may play a crucial role in TRPC3-mediated chronic pain (Wang T-Z. et al., 2023). In this scheme, PKG-I phosphorylation of TRPC3 (and TRPC6) increases channel activity, which augments calcium influx, and ultimately enhances neuronal excitability, potentially contributing to pain and pain-induced anxiety and depression.

### 4.5 Potential roles of other TRP channels in OA pain

In addition to the aforementioned channels, other TRP channel members also play significant roles in OA pain. The mechanically-induced ectopic pain observed in an MIA-induced OA pain model (and an experimental autoimmune encephalomyelitis model) was significantly alleviated when using a *TRPM2* knockout mouse model background (Zhang et al., 2020). These findings suggest that TRPM2 is crucial in mediating inflammatory and neuropathic pain mechanisms in these models. TRPM8, a major cold-sensing channel, was recently reported to be expressed in primary OA human chondrocytes (Halonen et al., 2023). Moreover, application of menthol, the main agonist of TRPM8, to OA chondrocytes increased the expression levels of several cartilage-degrading enzymes. This result suggests that TRPM8 may play a role in OA pathology. In addition, TRPC5 has been linked to inflammatory pain, including osteoarthritis (Khare et al., 2024). Collectively, these studies highlight the complex, networked, and multi-faceted roles of the TRP channel family in OA pain sensing and modulation.

In summary, TRP channels form an intricate regulatory network in pain perception. They function as both “sentinels” that directly convert noxious stimuli into electrical signals in neurons, and as “modulators” that amplify and sustain pain signals through the indirect release of mediators from non-neuronal cells. This interplay of direct and indirect mechanisms is particularly critical in chronic and inflammatory pain conditions, making TRP channels highly promising targets for the development of novel analgesic drugs.

## 5 Clinical research on drugs targeting TRP channels for treating OA

The clinical research performed to date on drugs targeting TRP channels for the treatment of OA are summarised in Table 3. Thus far, TRP channel drug development has predominantly focused on TRPV1. Both agonists and antagonists of TRPV1 are currently being investigated as potential treatments for OA pain. TRPV1 antagonists act directly on the TRPV1 receptor to attenuate pain signal transduction and the release of neuropeptides, thus inhibiting pain production. The analgesic effects of TRPV1 agonists, such as capsaicin, can be maintained for several weeks to several months, as these drugs act through desensitization, neuropeptide depletion, and the ablation of nociceptor terminals. However, post-treatment pain recovery has been reported following nociceptor nerve regeneration (Ragé et al., 2010). Because TRPV1 and TRPA1 are co-expressed in neurons, capsaicin-induced ablation of nociceptor terminals may also reduce TRPA1-mediated pain and inflammation (Bautista et al., 2005; Salas et al., 2009). Hence, TRPV1 agonists may have broader effects beyond modulating TRPV1 activity.

**TABLE 3:** Clinical Research on Drugs targeting TRP Channels for Treating OA.

Agonist/Antagonist	Drug	Clinical progress	Treatment	Number of patients	Effects	Refs
TRPV1 agonist	Civamide cream (0.025%)	NA	Civamide cream (0.025%; a 12-week, double-blind, 6-center study	113	Reduced pain	Altman et al. (1994), McCleane (2012)
TRPV1 agonist	Capsaicin and glyceryl trinitrate	​	placebo, capsaicin (0.025%), glyceryl trinitrate (1.33%), or capsaicin (0.025%) and glyceryl trinitrate (1.33%); four times daily for 6 weeks	167	analgesic effect	Persson et al. (2018)
TRPV1 agonist	Civamide cream (0.075%)	NA	civamide cream (0.075% or 0.01%)	695	​	Schnitzer et al. (2012)
TRPV1 agonist	CNTX-4975	Phase II	Intra-articular injection of CNTX-4975 (0.5 mg or 1.0 mg) or a placebo; for 24 weeks	175	Reduced pain	Stevens et al. (2019)
TRPV1 antagonist	NEO6860	Phase I	NEO6860 (500 mg), naproxen (500 mg), or placebo; twice daily for 1 day	54	analgesic effect	Arsenault et al. (2018)
TRPV1 antagonist	Mavatrep (JNJ-39439335)	Phase I	mavatrep (10, 25, or 50 mg) or placebo; once a day	24	Reduced pain	Manitpisitkul et al. (2018)
TRPV1 antagonist	AZD1386	Phase II	AZD1386 (30 mg or 90 mg) or placebo; 4 weeks	191	No significant pain reduction	Miller et al. (2014)

A four-week, double-blind, randomized, multicenter study revealed that OA patients experienced significant pain relief (compared with a placebo) after local application of capsaicin cream (Zhang and Li Wan Po, 1994; Altman et al., 1994). Aside from a transient burning sensation at the treatment site, no other side effects were reported. The effects of topical treatments on OA pain were further investigated in a randomized, double-blind, placebo-controlled study (McCleane, 2012; Persson et al., 2018). Here, the participants were divided into four treatment regime groups (Group A, 0.025% capsaicin; Group B, placebo; Group C, 1.33% glyceryl trinitrate; Group D, 0.025% capsaicin combined with 1.33% glyceryl trinitrate), and the study included a 6-week follow-up. The results of this study provided further confirmation that local capsaicin has analgesic effects on OA pain. Interestingly, the analgesic effect was more pronounced in the capsaicin and glyceryl trinitrate combined treatment group (Group D) than in the capsaicin only treatment group (Group A). Another TRPV1 agonist, civamide (at 0.075%), which operates similarly to capsaicin, has also been shown to be effective in relieving pain in OA patients after continuous treatment for 1 year (Schnitzer et al., 2012). Importantly, local application of civamide does not lead to systemic absorption, reducing the potential for toxicity.

The synthetic trans-capsaicin CNTX-4975 is a highly potent TRPV1 agonist, and it has been demonstrated to reduce OA-related pain. In a 24-week randomized, double-blind, placebo-controlled clinical trial, 175 patients with moderate to severe OA pain who had previously failed treatment were treated with two doses of CNTX-4975 (0.5 mg or 1.0 mg) or a placebo (Stevens et al., 2019). The patients who received 1 mg of CNTX-4975 showed a significant improvement in the primary efficacy endpoint, the daily WOMAC pain score between baseline and week 12, in comparison with patients in the placebo group. The adverse events reported post-treatment included joint pain, upper respiratory tract infection, increased liver enzymes, joint effusion, and OA. However, there were no differences in the frequency or severity of these events between the CNTX-4975 and placebo groups. In 2018, CNTX-4975 received a fast-track designation from the U.S. FDA for treating KOA pain.

In a phase II clinical trial investigating the efficacy of the TRPV1 antagonist NEO6860 for treating OA, 54 KOA patients were randomly assigned to receive either NEO6860 (500 mg, twice daily), placebo, or naproxen (Arsenault et al., 2018). The primary endpoint of this randomized, double-blind, three-period crossover study was a reduction in pain intensity (PI) on the Numeric Rating Scale following a stair climbing exercise performed 8 hours post-treatment. While the results revealed that NEO6860 did not significantly outperform the placebo (in terms of the primary endpoint), patients did report some analgesic effects (with no effect on body temperature or heat pain perception). Hence, further research is warranted to explore the potential of NEO6860 in other pain relief applications.

In a phase I clinical trial, the TRPV1 antagonist JNJ-39439335 (mavatrep) was reported to significantly alleviate the pain associated with stair climbing in KOA patients (Manitpisitkul et al., 2018). This double-blind, randomized, placebo-controlled, sequential group, multiple ascending-dose study also evaluated the tolerability, pharmacokinetics, and pharmacodynamics of mavatrep in KOA patients. Importantly, mavatrep demonstrated good tolerability when administered in doses ranging from 2 to 50 mg.

In a separate clinical study, researchers investigated the TRPV1 antagonist AZD1386 (Miller et al., 2014). In contrast to the findings with JNJ-39439335, AZD1386 did not significantly improve pain outcomes (when compared with placebo).

For future studies, it is crucial that the mechanism of potent TRPV1 agonists be distinguished from that of TRPV1 antagonists. Agonists such as capsaicin, trans-capsaicin, and civamide act as “molecular scalpels”, inducing calcium-dependent defunctionalization and ablation of the nociceptive nerve terminals that express TRPV1. Because these terminals co-express a multitude of other pain-transducing channels (e.g., TRPA1, voltage-gated sodium channels), this process effectively creates a functional multi-channel blockade, resulting in profound and long-lasting analgesia. In contrast, TRPV1 antagonists provide a selective and reversible blockade of a single pathway, leaving the neuron functionally intact, and this explains their more limited analgesic efficacy profile.

## 6 Conclusion

In summary, TRP channels play diverse roles in the pathogenesis of OA. Research has revealed their involvement in several different aspects of OA chondrocyte function, including gene expression, metabolism, apoptosis, and ferroptosis. These complex effects contribute to maintaining chondrocyte homeostasis in OA. TRP activity is also important in synovial macrophages, fibroblasts, neutrophils, and other cell types involved in OA pathogenesis. Importantly, multiple TRP activities have been shown to be closely associated with OA pain. Recent clinical investigations have explored new analgesic drugs that target the TRPV1 channel, which has demonstrated analgesic activity. Together, these findings should prompt further exploration of the unknown mechanisms by which TRPs contribute to OA pathogenesis, with the ultimate goal of alleviating the suffering of OA patients.
